# Contexts of urgency may go beyond emotion

**DOI:** 10.3389/fpsyt.2024.1412001

**Published:** 2024-09-12

**Authors:** Matthew V. Elliott, Oliver P. John, J. D. Allen, Sheri L. Johnson

**Affiliations:** Department of Psychology, University of California, Berkeley, Berkeley, CA, United States

**Keywords:** emotion, impulsivity, psychopathology, self-control, urgency

## Abstract

**Introduction:**

Urgency has been defined as the tendency towards rash speech and behavior in the context of emotion. Measures of Urgency have been found to have robust predictive power for psychopathologies and problematic behaviors. In the current study, we question whether emotions are unique drivers of urgency, or if emotions are potent exemplars of contexts that lead to rash speech and behavior. The Emotion Specific model and the Broader Contexts model correspond with these two conceptualizations of urgency, and they frame our pre-registered hypotheses.

**Methods:**

Participants from two well-powered samples (*n* = 600,*n* = 588) completed 9 modified items from the Urgency and Positive Urgency scales to assess rash responses in each of four contexts – “Upset,” “Excited,” “Tired,” and “Hungry” – and a fifth “In General” set. After data cleaning, we used principal components analysis to construct a unidimensional, 4-item set that was applied to capture impulsive behavior across the five contexts.

**Results:**

We found that this research tool, called the Contexts of Impulsive Behaviors (CIBS), replicated in the second dataset, and it had adequate internal reliability in both samples. Although the Emotion Specific model was supported by the fact that the Upset context had a greater mean and greater variance than the Tired and Hungry contexts, most results supported the Broader Contexts model. That is, CIBS contexts were highly intercorrelated, and bivariate correlations with psychopathology were not significantly different across contexts. In partial correlations, effects of the Upset and Excited contexts were partially or fully statistically mediated by the Tired and Hungry contexts.

**Discussion:**

These findings suggest that emotions are potent contexts for impulsive behaviors. At the same time, those with high urgency are vulnerable to impulsivity in other contexts, such as fatigue and hunger, that challenge the regulatory functions of the prefrontal cortex. Limitations, future directions, and clinical implications are discussed.

## Introduction

In recent decades, statistical and theoretical advances have converged on the prevailing idea that impulsivity is not a unitary construct, but rather a set of separable dimensions (1, 2). One of the most replicable and influential multidimensional models of impulsivity suggested that some people have a trait-like propensity to act impulsively when experiencing strong emotions, as captured on the factor-analytically derived UPPS-P scale (2, 3). The (Negative) Urgency (NU) and Positive Urgency (PU) scales from the UPPS-P are comprised of items designed to capture tendencies toward rash speech and behavior prompted by negative and positive emotions, respectively. Beyond their inherent relevance to personality science, Urgency measures have been influential in clinical psychology because of their robust predictive validity for a broad range of internalizing psychopathologies – including depression, disordered eating, and suicidal ideation (4–8) – and externalized maladaptive behaviors – including aggression, risky sexual behaviors, gambling problems, non-suicidal self-injury, suicidal actions, and substance abuse (9–15). Urgency is now considered a transdiagnostic risk factor for psychopathology (16, 17).

Based on the clinical validity of urgency measures, much effort has been invested to understand *how* emotional and self-regulatory systems operate and interact among persons with severe urgency. One logical starting point is the idea that urgency is a behavioral manifestation of heightened emotional reactivity (i.e., generating emotions more strongly on average) (18, 19). Laboratory investigations of this model, however, have not provided strong support. Several studies have found that individuals with higher urgency do not show greater subjective or psychophysiological responses to standardized stress or positive mood inductions (20–23). Other work has demonstrated that effects of urgency remain when controlling for neuroticism, a trait-based operationalization of stronger emotional reactivity (24, 25). While heightened emotional reactivity is likely a factor that can compound the adverse effects of urgency, it does not seem to be requisite for its emergence.

Another prominent model posits that urgency stems from deficits in inhibitory control circuitry (26). Although a meta-analysis showed that inhibitory control task performance relates to urgency in clinical samples with primary inhibitory control deficits, such as those with traumatic brain injury and attention deficit/ hyperactivity disorder (27, 28), these correlations are weak in student and community samples (29). Theory, though, has emphasized that inhibitory control would be of particular importance in the context of heightened emotion (26, 30). Consistent with this idea, two studies using “emotional” stop-signal tasks found that Urgency correlated with response inhibition on trials with emotional stimuli but not on trials with neutral stimuli (31, 32). A third study found that among participants with higher Urgency, even minor increases in pupil dilation – a physiological marker of arousal – predicted a decay in accuracy on an anti-saccade task (23). In addition to these findings in response inhibition tasks, researchers using risky decision-making tasks have found that sexual cues (33), pharmacological manipulation of physiological arousal by yohimbine (34), and laboratory stress induction (35) all led to more risky behavior for participants with more severe Urgency. Parallel findings have been observed in fMRI studies (see 36, 37), in that Urgency has been tied to differential profiles of cognitive control circuitry during response inhibition tasks involving emotional stimuli (38–40). Taken together, these studies suggest that one mechanism of urgency may be the fragility of inhibitory control systems when they are pushed outside of “cold” (i.e., calm) states into states of high arousal.

If the key ingredient for urgency is fragile inhibitory control circuitry, then other contexts beyond emotions and physiological arousal that have been shown to affect those circuits may lead to similar patterns of unconstrained behavior. Fatigue due to lack of sleep is one plausible candidate. Neuroimaging studies have shown that experimental induction of sleep deprivation causes reduced activation of inhibitory control brain networks and poorer cognitive task performance (41–43). Weaker functional connectivity between prefrontal cortex and amygdala, a circuit thought to exert inhibitory control over emotional responses, has also been found following sleep deprivation (44). Hunger is another physiological context that can affect prefrontal functioning. A recent systematic review found significant decrements in inhibitory control task performance following experimental fasting in 68% of published studies (45). Of the cognitive domains reviewed, inhibitory control was the most consistently impaired after fasting. Stated differently, if urgency reflects the influence of emotion on an already fragile inhibitory control circuitry, one might expect that other challenges to this circuitry could similarly prompt rash behavior in those who demonstrate urgency.

In the current study, we aim to test the idea that a fuller conceptualization of urgency may go beyond the traditional focus on emotional contexts of impulsive action. That is, although emotion is a common and potent context of decays in inhibitory control for those with urgency, fragility in inhibitory control circuitry may also be vulnerable to other contextual challenges, such as fatigue and hunger. We frame this line of inquiry using two guiding questions:

1. Are emotion contexts of impulsive behaviors distinct from broader challenges to inhibitory control, such as hunger and fatigue?
2. Is a tendency toward impulsive behavior that is contextualized by emotions a stronger predictor of psychopathology than tendencies toward impulsive behavior in broader physiological contexts, such as hunger and fatigue?

To investigate these questions, we define two contrasting models that yield our pre-registered hypotheses. The Emotion Specific model predicts that impulsivity in the context of emotion is more common than and statistically distinct from trait-like tendencies to act impulsively when tired or hungry. The Emotion Specific model also predicts that emotion-context impulsivity confers greater risk for psychopathology than do trait-like tendencies to act impulsively when tired or hungry. Conversely, the Broader Contexts model predicts that trait-like vulnerabilities to emotion and broader physiological contexts will be similarly common and highly correlated. The Broader Contexts model also predicts that emotion-context impulsivity and impulsivity in broader physiological contexts will confer similar risk for elevated psychopathology.

To test the Emotion Specific and Broader Context models, we create a set of items that are modified from the Urgency and Positive Urgency scales to allow for tests of emotional and broader physiological contexts. We do not intend to improve upon nor displace existing Urgency measures. Instead, we validate this research tool so that it can be used to conduct our specific pre-registered analyses to test the levels of endorsement and variability for each context, and the correlations of the contexts with psychopathology. We consider bivariate correlations of impulsivity in each context with psychopathology, and then we consider whether impulsivity in emotion contexts shows unique correlations when controlling for impulsivity in physiological contexts. In assessing correlations with psychopathology, we focus on depression as an internalizing syndrome that has been shown to relate to Urgency and aggression as an externalizing dimension that has been shown to relate to Urgency (46–48).

## Materials and method

The pre-registration for this study is available: (https://aspredicted.org/V2Y_G5Z). Both studies were approved by the university Committee for the Protection of Human Subjects. All participants completed informed consent before study procedures.

### Participants and procedures

The data used in the current investigation were collected as part of two larger studies that recruited from an undergraduate research participation program at a large public university between 09/2021 – 04/2022. By this time, the university had returned to primarily in-person classes following the COVID-19 lockdown. To test the reproducibility of our findings, the data from these two samples are kept separate in all data cleaning and analysis steps. We refer to Sample 1 and Sample 2 to differentiate the samples. The data collection and analysis methods are highly parallel across these two samples, and we clearly note where any differences exist.

Recruitment was conducted via online advertising to all students enrolled in Psychology classes. Students were excluded from participation if they were under the age of 18. Participation was fully remote. Participants received partial course credit as compensation for their participation. We expected that we would need to check the unidimensionality of the items to be used in our research. Thus, we aimed for final samples of 400 participants because factor analyses are very stable and replicable at these sample sizes. These sample sizes also provide ample power for our correlational analyses. We expected at least small-to-medium sized correlations (i.e., above .20). Even with half the sample size (*N* = 200), G*Power indicates that we can detect bivariate r’s and partial r’s of .25 with power of .80 (49). Even more important, the replication design of this research provides an explicit check of the assumption that we have sufficient power to address the present research questions.

In both samples, pre-screening procedures were used to attempt to oversample for low base-rate behaviors that might reflect problems with self-control of different forms. Sample 1 pre-screening focused on students who reported a history of behaving in a sexually coercive manner on the Sexual Coercion in Intimate Relationships Scale (50), and Sample 2 pre-screening focused on students who reported a history of suicidal ideation on the Columbia Suicide Severity Rating Scale (51). Students who met these pre-screening criteria were sent an email inviting them specifically to participate in the study. To allow for recruitment across the full range of impulsivity, study participation was also fully available for other students in the research participation program. Our pre-screening attempts were not successful: base rates of sexual coercion were too low to support analyses. 99.3% of participants scored between 0 and 1 on the 6-point Sexual Coercion in Intimate Relationships Scale. In a deviation from our pre-registered analysis plan, given the key goal of testing the reproducibility of our results and the low base rates of behaviors endorsed on both scales, we do not include analyses of the sexual coercion or suicidal ideation measures here. Analyses of the Three Factor Impulsivity scale with suicidal ideation are provided in a previous publication (52).

Sample 1 had a total size of 598, and Sample 2 had a total size of 586.

### Measures

#### Initial item set to measure broader contexts of impulsivity

To represent a broad range of content related to urgency, we initially selected nine item probes from the Urgency and Positive Urgency measures (2, 53). To select items, two impulsivity researchers reviewed the content of each item, and considered applicability to the hunger or tired context. As an example, items concerning “Hard to resist acting on feelings” and “When rejected say things later regret “ are hard to tailor to the hunger and tired context, and so were omitted. Items concerning “Have trouble resisting cravings” may have a unique meaning in the context of hunger, and so were omitted. We modified the selected probes to remove emotional contexts specified in the original items (e.g., “I often make matters worse because I act without thinking when I am upset” was modified by removing the “when I am upset” specifier). Instead, participants were instructed to answer each of the 9 Urgency probes while recalling times when they experienced each of four contexts – “Upset,” “Excited,” “Tired,” and “Hungry.” We also included a fifth, “General,” item set that asked how true the statements were for their life “in general” without regard to particular contexts. To be consistent with work on the Three-Factor Impulsivity Scale (54), participants rated each of these 45 self-report items on a 5-point Likert scale, ranging from 1 (I agree a lot) to 5 (I disagree a lot).

#### Three factor impulsivity index

To validate our Contexts of Impulsive Behaviors research tool against well-validated measures of impulsivity, we collected the Three Factor Impulsivity Index (TFII; 54). Here, we focus on the Feelings Trigger Action and Lack of Follow Through scales, which are well-validated and have replicable structures using factor analysis (15, 55).

The Feelings Trigger Action (FTA) scale contains 26 items from the Negative Urgency scale (e.g., “I often make matters worse because I act without thinking when I am upset”; 2), the Positive Urgency scale (e.g., “When I am really excited, I tend not to think of the consequences of my actions”; 53), and Reflexive Reactions to Feelings scale (e.g., “I generally act on my feelings instantly”; 54). The Lack of Follow Through (LFT) scale is composed of 19 items from the Distractibility and Lack of Perseverance scales (2, 54), for example, “Its hard for me to keep my mind from wandering.”

The Three Factor Impulsivity Index items used 5-point Likert scales from 1 (I agree a lot) to 5 (I disagree a lot). While FTA items measure impulsive behaviors in the context of strong emotions, LFT items do not include references to emotions. The LFT scale has shown strong psychometric properties and is statistically distinct from measures of Urgency (54, 55). For these reasons, LFT has been used as a conceptually-adjacent, control comparison to demonstrate specificity of effects in studies of Urgency (23, 56). We included LFT for this purpose. Multiple studies have shown that FTA scores are more robustly related to early adversity (54), depression (46), multiple facets of suicide risk (7, 52), suicide attempt history (15), and internalizing symptoms (55) than the LFT scale. LFT has also been validated against psychopathology indices, including ADHD (57). After reverse-scoring negatively keyed items, FTA and LFT scores were calculated by averaging across their 26 and 19 items, respectively.

#### Center for epidemiologic studies depression scale – revised

The Center for Epidemiologic Studies Depression Scale-Revised (CESD-R) was used to capture current depressive symptom severity (58). The CESD-R consists of 20 items covering affective, cognitive, and somatic symptoms of depression, for example, “I lost interest in my usual activities.” The CESD-R is internally reliable and has strong convergent and divergent validity (58, 59). Participants are asked to rate the frequency of experiencing each symptom over the past week on a 4-point Likert scale ranging from 1 (Rarely or none of the time, Less than 1 day) to 4 (Most or all of the time, 5–7 days). After reverse-scoring negatively keyed items, CESD-R depression scores are calculated by averaging across the 20 items.

#### Bryant aggression questionnaire

The Bryant Aggression Questionnaire is a short form of the Buss-Perry Aggression Questionnaire (AQ) designed to cover tendencies toward anger and aggressive behavior, for example “I have threatened people I know” (60). The BAQ was developed using factor analysis to identify four 3-item subscales, and has shown more robust psychometric characteristics than the original AQ. Participants are asked to rate how characteristic each statement was for them on a 5-point Likert scale ranging from 1 (Extremely uncharacteristic of me) to 5 (Extremely characteristic of me) and subscales are scored as the mean of the 3 items. Here, we focused on the three subscales previously shown to be correlated with Urgency: Physical Aggression, Verbal Aggression, and Anger subscales, omitting the Hostility subscale (61). As all 3 subscales have been found to correlate with Urgency, and we had no differential hypotheses for the three subscales, we computed a total BAQ scale by averaging the three subscales.

### Analysis plan

#### Data cleaning

As stated in our pre-registration, we excluded participants using equivalent criteria in the Sample 1 and Sample 2. Participants were excluded from data analysis if they met one or more of the following criteria: 1) Failed one or more “catch” trials (e.g., “On this item, respond with Never”), 2) completed the full survey in fewer than 500 seconds, 3) did not answer (i.e., had missing data) one or more items on the 45-item Broader Contexts of Impulsivity Item Set, or 4) demonstrated careless responding on the Broader Contexts of Impulsivity Item Set by either providing the same response to every item or having average scores for both positively and negatively keyed items below 2.0 or 4.0 on the 1–5 response scale. Participants with missing data on the LFT, CESD-R or Bryant, whose data was otherwise acceptable, were excluded from the analyses that used those respective measures only.

#### Sample 1: construction of the Contexts of Impulsive Behaviors

In Sample 1, we first checked whether the 9 item probes selected from the Urgency and Positive Urgency measures formed a unidimensional item set in each context. Using principal components (PC) analysis, the 9 items were not unidimensional in any of the 5 contexts. Instead, they measured more than one factor, as indicated by both the scree test and parallel analysis. As an example, we have included the results for the Hungry context, showing the loadings of the 9 candidate items on the three unrotated components, in **Appendix B** in the online materials. Specifically, items 2, 1, 5, 7, and 9 all showed clear simple structure: they all loaded above .70 on the first principal component, and none had a loading of .35 or above on the other two components. In contrast, the remaining four items (8, 4, 3, and 6) were factorially complex: They all had loadings below .70 on the first principal component as well as substantial loadings on at least one of the two other components. In fact, one of these items (i.e., “I feel like I cant stop myself from going overboard”) loaded only .11 on the first unrotated component, which captures the core Urgency factor assumed to underlie all the items. Several other items had substantial secondary loadings on the second or third component (e.g., “Others are shocked or worried about the things I do”), indicating that they measured other aspects of behavior in addition to a single dimension of individual differences in impulsivity.

These issues with dimensionality were apparent not only in the Hungry context but also in the other three contextualized ratings as well as in the General ratings. Because we needed a unidimensional item set as a tool for our pre-registered analyses, we selected only those item probes that showed simple structure, measuring only the intended primary factor and did so consistently in the four context-specific instructions as well as the General instructions.

The results of the PC analyses for all 9 items and in all 5 contexts are summarized in **Table 1**. The results were clear and consistent across all the contexts. The five items shown in the upper part of **Table 1** all had average loadings across the 5 contexts above .70, had loadings of at least.68 in every context, and showed simple structure (i.e., no cross-loadings above .35). In contrast, none of the 4 remaining items in the lower part of **Table 1** met these three conditions. Finally, when we reviewed this empirically selected set of 5 items, we noted that it included two items that were highly redundant, both asking about actions one later regrets. Thus, as indicated in **Table 1**, we retained only the shorter and simpler item of the two (i.e., “I will often say things that I later regret”), resulting in a 4-item research tool.

**Table 1:** Sample 1: loadings on the first unrotated principal component for the 9 candidate Items in four specific contexts and in general.

Item #	Item Text	Upset	Tired	Hungry	Excited	General	Average
Items showing simple structure and average loadings across contexts above .70							
2	*I will often do things I later regret in order to make myself feel better now.*	**.82**	**.81**	**.82**	**.84**	**.84**	**.82**
1	**I often make matters worse because I act without thinking.**	**.87**	**.83**	**.82**	**.82**	**.75**	**.82**
5	**I will often say things that I later regret.**	**.79**	**.78**	**.74**	**.74**	**.76**	**.76**
7	**I think of the consequences of my actions. (R)**	**.80**	**.76**	**.79**	**.75**	.68	**.75**
9	**I cant seem to stop what I am doing even though it is making me feel worse.**	**.73**	**.73**	**.76**	**.71**	**.71**	**.73**
Factorially complex items with average loadings across contexts below .70							
6	*Others are shocked or worried about the things I do.*	**.71**	.62	.66	**.76**	.70	.69
3	*I do not have trouble controlling my impulses. (R)*	.67	.66	.49	.56	.66	.61
4	*I have trouble resisting my cravings (for food, cigarettes, etc).*	.56	.55	.54	.49	.56	.54
8	*I feel like I cant stop myself from going overboard.*	.19	.13	.11	.00	.04	.09

The item text of the four items selected in Sample 1 are shown in bold. The items not selected are shown in italics. Loadings above .70 are shown in bold.

These steps were completed using only the sample from Sample 1 in an exploratory (i.e., data-driven) analysis. Then, these 4 items were examined again in Sample 2 to test for replicability (results described below). Following the selection and validation procedures, we refer to the final item set as the Contexts of Impulsive Behaviors (CIBS) research tool. **Table 2** contains the full set of 20 items, that is, each of the 4 final impulsivity items rated in each of the 5 contexts.

**Table 2:** Final item set of the Contexts of Impulsive Behaviors.

Please rate how true the following statements are for your life in general.	
1.1	I often make matters worse because I act without thinking. [NU]
1.2	I will often say things that I later regret. [NU]
1.3	I think of the consequences of my actions. (R) [PU]
1.4	I can’t seem to stop what I am doing even though it is making me feel worse [NU]
Now, we want you to think about times when you are feeling upset. Please rate how true the following statements are for you when you are upset	
2.1	I often make matters worse because I act without thinking.
2.2	I will often say things that I later regret.
2.3	I think of the consequences of my actions. (R)
2.4	I can’t seem to stop what I am doing even though it is making me feel worse
Now, we want you to think about times when you are feeling very excited. Please rate how true the following statements are for you when you are very excited.	
3.1	I often make matters worse because I act without thinking.
3.2	I will often say things that I later regret.
3.3	I think of the consequences of my actions. (R)
3.4	I can’t seem to stop what I am doing even though it is making me feel worse.
Now, we want you to think about times when you have not had enough sleep. Please rate how true the following statements are for you when you have not had enough sleep.	
4.1	I often make matters worse because I act without thinking.
4.2	I will often say things that I later regret.
4.3	I think of the consequences of my actions. (R)
4.4	I can’t seem to stop what I am doing even though it is making me feel worse.
Now, we want you to think about times when you are feeling hungry. Please rate how true the following statements are for you when you are hungry.	
5.1	I often make matters worse because I act without thinking.
5.2	I will often say things that I later regret.
5.3	I think of the consequences of my actions. (R)
5.4	I can’t seem to stop what I am doing even though it is making me feel worse.

Four items were adapted from measures of Positive Urgency [PU] and Negative Urgency [NU] and applied to Five Contexts: Upset, Excited, Tired, Hungry, and General.

#### Sample 2: replicating the unidimensional Contexts of Impulsive Behaviors

As described above, the 4-item CIBS was developed in the Study 1 sample. Thus, we used Sample 2 to test whether our item selection procedure replicated and resulted in a unidimensional item set. Specifically, to evaluate unidimensionality, we report (a) the loadings of the 4 items on the first unrotated principal component (expected to be .70 or above), (b) the percent of variance accounted for by the first principal component (expected to be greater than 50%), and (c) the fit statistics for the unidimensional model tested with confirmatory factor analysis (CFA); good fit is indicated by CFI and TLI values of .90 and above, RMSEA values of .08 and below, and SRMR values of .05 and below. For each CFA, we chose to fix item 1 to the latent factor for identification since it had the highest average loadings on the first, unrotated principal component in Sample 1 (**Table 1**).

In addition, we evaluated the internal reliability of the resulting unidimensional item set using Cronbach's alpha. Adequate internal reliability (α > 0.75) was required prior to continuing with our pre-registered analyses. Going beyond the pre-registration, we calculated the average inter-item correlation (AIC) of each context as a second indicator of internal consistency. AIC is not affected by the number of items in the scale; therefore, it can be a better index of internal reliability for brief self-report scales (62). We set an AIC of greater than 0.4 as our standard of evidence for high internal consistency in our unidimensional item set.

#### Contexts of Impulsive Behaviors characteristics and predictive validity

After replicating the CIBS and examining its reliability, we conducted our pre-registered analyses, designed to compare evidence for and against the Emotion Specific and the Broader Contexts models of urgency. We conducted analyses identically in Sample 1 and Sample 2. All tests were two-tailed with alpha = 0.05. We clearly state when analyses were not pre-registered and include justification for adding them.

To begin, we consider whether emotion-context impulsivity was more common, or showed more pronounced individual differences than did impulsivity in other contexts. We conducted paired samples *t* tests to test for significant differences among the means of the five CIBS contexts, as well as LFT. Going beyond the pre-registration, we also calculated Cohen's *d* to provide effect sizes for these differences of sample means. We used paired Pitman-Morgan tests to test whether the sample variances of these contexts differed significantly. To condense the number of tests, we deviated from our pre-registration and only tested differences among contexts with adjacent means and variances. To bolster these tests and utilize our replication design, we also calculated a rank-order correlation of the means and variances of the contexts.

Significantly higher mean scores and variances for emotion contexts than the other contexts would be consistent with the Emotion Specific model, reflecting that emotion is a context that is separable and with more power to evoke impulsive behaviors. Findings that emotion contexts, but not broader physiological contexts, would have significantly different means and variances compared to the LFT scale, would also be consistent with the Emotion Specific model. In contrast, finding that broader CIBS contexts are as or more common than CIBS emotion contexts would be consistent with the Broader Contexts model. Findings that emotion and broader CIBS contexts would have significantly different means and variances compared to the LFT scale, would also be consistent with the Broader Contexts model.

We also considered the intercorrelations of the CIBS contexts. We did not pre-register hypotheses for this analysis; however, relatively low correlations between the CIBS emotion contexts and broader physiological CIBS contexts could be interpreted as evidence in favor of the Emotion Specific model. On the other hand, comparable intercorrelations within and between these context types could be interpreted as evidence in favor of the Broader Contexts model.

Next, we examined the bivariate correlations of CIBS and LFT with psychopathology. Our choice of correlation coefficient matched the distributional properties of each psychopathology measure. To compare the strengths of the magnitude of correlations for the CIBS contexts and LFT on psychopathology, we used bootstrapping (1000 random samples with replacement) to build 95% confidence intervals around each correlation coefficient. Correlation coefficients were considered to be significantly different from each other if the 95% confidence intervals did not overlap one another. The Emotion Specific model predicted that emotion-context impulsivity would have significantly larger effect sizes with psychopathology than impulsivity in broader physiological contexts and LFT. The Broader Contexts model predicted that bivariate correlations of CIBS emotion contexts and CIBS broader contexts on psychopathology would all be significantly larger than LFT, and they would not be significantly different from one another.

Although the bivariate correlations of CIBS with psychopathology scales were of primary interest, we also aimed to understand the unique (i.e., non-shared) variance of the CIBS contexts on psychopathology. In our pre-registration, we stated that we would use multivariate linear models to examine the effects of the CIBS emotion contexts controlling for the broader physiological CIBS on psychopathology scales. To provide comparable information, we ultimately decided to present partial correlations because they are simpler tests of unique effects on psychopathology. The Emotion Specific model predicted that CIBS emotion contexts would significantly correlate with psychopathology when controlling for impulsivity in broader contexts; in contrast, the Broader Contexts model predicted that the unique effects of CIBS emotion contexts on psychopathology would be statistically mediated by impulsivity in broader contexts.

Going beyond the pre-registration, we tested whether impulsivity in emotion and broader contexts predicted psychopathology when controlling for the “General” CIBS. We included this analysis because of the sizable direct effects of the “General” CIBS on psychopathology. In the case that effects of emotion contexts, but not broader physiological contexts, remained significant when controlling for the “General” CIBS, this analysis would provide evidence for the Emotion Specific model. In the case that all effects of CIBS emotion contexts and CIBS broader physiological contexts on psychopathology remained significant when controlling for the “General” CIBS, this analysis would provide evidence for the Broader Contexts model. We also tested whether impulsivity when tired or hungry predicted psychopathology when controlling for emotion-context impulsivity. Last, we tested the impact of controlling for LFT, which has frequently been considered in previous studies of emotion-context impulsivity and psychopathology. Both models predicted the same outcome – that CIBS would correlate significantly with psychopathology when controlling for LFT.

## Results

### Data cleaning

In Sample 1 (S1), quality assurance led to the exclusion of 196 participants: 43 for skipping one or more items on the Broader Contexts of Impulsivity Item Set, 26 participants for completing the survey too quickly, 119 participants for incorrectly answering one or more “catch trials,” and 8 participants for other forms of careless responding. In Sample 2 (S2), quality assurance led to the exclusion of 184 participants: 43 participants for skipping one or more items on the Broader Contexts of Impulsivity Item Set, 31 participants for completing the survey too quickly, 98 participants for incorrectly answering one or more “catch trials,” and 12 participants for other forms of careless responding. After completing these data cleaning procedures, Sample 1 had a final size of 402, and Sample 2 had a final size of 402. Young women comprised the majority of both samples (S1: M_age_ = 20.5, SD_age_ = 2.18, 76% women; S2: M_age_ = 20.7, SD_age_ = 2.86, 76% women). Asian/Asian American (S1: 46%, S2: 51%), White/European American (S1: 31%, S2: 28%) and More Than One Race/Other (S1: 17%, S2: 16%) were the most common racial identities represented in the two samples. **Table 3** summarizes the sociodemographic characteristics of the final samples.

**Table 3:** Sociodemographic characteristics of the final samples.

Sample characteristics	Sample 1		Sample 2	
	*n*	%	*n*	%
Final sample size	402		402	
Gender				
Woman	304	75.6	304	75.6
Man	91	22.6	92	22.9
Non-Binary	5	1.2	6	1.5
Declined to respond	2	0.5	0	0
Race				
American Indian/ Alaska Native	3	0.7	2	0.5
Asian/Asian American	183	45.5	204	50.7
Black/African American	4	1.0	3	0.7
More than one race/Other	68	16.9	65	16.2
Native Hawaiian/ Pacific Islander	1	0.2	0	0
White/European American	126	31.3	111	27.6
Declined to respond	17	4.2	17	4.2
Ethnicity				
Hispanic or Latina/o	76	18.9	80	19.9
Not Hispanic or Latina/o	311	77.4	298	74.1
Other	7	1.7	15	3.7
Declined to respond	7	1.7	9	2.2
School Year				
Freshman	69	17.2	62	15.4
Sophomore	54	13.4	57	14.2
Junior	146	36.3	147	36.6
Senior	125	31.1	128	31.8
Graduate/Other	8	2.0	8	2.0

### Sample 2: replication of the 4-item contexts of impulsive behaviors

To test whether our item selection procedure had successfully resulted in a unidimensional research tool for each of the 5 contexts, we used the data from Sample 2 as a replication sample. **Table 4** reports the results. First, the factor loadings of the 4 items on the first unrotated factor were all substantial in size and averaged .81 across the 4 items as well as the 5 contexts. In fact, all but one of these 20 item loadings were above .70, indicating the items shared more than 50% of their variance with the impulsivity factor we intended to measure. Second, **Table 4** reports the percent of variance accounted for by the first principal component, which averaged 64% across the 5 contexts, indicating that this principal dimension captured almost 2/3 of the total variance. Third, CFAs tests showed that the single-factor model generally fit the data very well, as summarized in **Table 4**: The CFI and TLI values for the 5 contexts were all above .94, the SRMR values were all below .05, and all but one of the 5 RMSEA values were below .08. On all indicators considered, then, we found considerable evidence that the CIBS measured a unidimensional construct in an independent replication sample.

**Table 4:** Testing the unidimensionality of the Contexts of Impulsive Behaviors in sample 2: principal component loadings for all 4 items, % of total variance explained, and confirmatory factor analysis fit statistics for the single-factor model in five contexts.

	Upset	Tired	Hungry	Excited	General	Mean across contexts
PC1 Loadings						
Item 1	.87	.86	.84	.85	.85	.85
Item 2	.86	.84	.80	.83	.83	.83
Item 3	.82	.81	.79	.76	.74	.78
Item 4	.75	.73	.76	.78	.66	.78
Mean Across Items	.83	.81	.80	.81	.77	.81
Total Variance Explained	68%	66%	63%	65%	60%	64%
CFA Fit Statistics						
CFI	1.00	1.00	0.99	0.98	1.00	0.99
TLI	1.00	1.00	0.97	0.94	1.00	0.98
RMSEA	.000	.000	.074	.115	.000	.038
SMR	.002	.004	.019	.027	.014	.013

CFA, Confirmatory Factor Analysis; CFI, Comparative Fit Index; PC1, First unrotated principal component; RMSEA, Root Mean Square Error of Approximation; SMR, Standardized Root Mean Square Residual; TLI, Tucker-Lewis Index.

As shown in **Table 2**, the final four probes included, “I often make matters worse because I act without thinking,” “I will often say things that I later regret,” “I think of the consequences of my actions [reverse coded],” and “I cant seem to stop what I am doing even though it is making me feel worse.” The Cronbach's alpha and average interitem correlations (AIC) for the 4-probe CIBS provided evidence of strong internal consistency (all αs > 0.75, all AICs > 0.40) for all five contexts. As shown in **Table 5**, the internal consistency estimates from Study 1 were very closely replicated in Sample 2: the five alpha values were within .02 of one another and the AIC values were within .04 of one another. Internal consistency of the existing measures – CESD-R, BAQ, FTA, and LFT – were strong (all αs > 0.80, **Table 5**).

**Table 5:** Means, standard deviations, and alpha reliabilities for the Contexts of Impulsive Behaviors in five contexts, the lack of follow through scale, the CESD-R depression scale, and the bryant aggression questionnaire in sample 1 and sample 2.

	Mean		Standard Deviation		Cronbach's Alpha		Average Interitem Correlation	
	S1	S2	S1	S2	S1	S2	S1	S2
CIBS								
Upset	2.70	2.65	1.15	1.11	.85	.84	.60	.57
Tired	2.50	2.43	1.08	1.06	.82	.82	.54	.54
Hungry	2.32	2.26	1.04	1.00	.82	.81	.54	.51
Excited	2.02	1.99	0.90	0.93	.80	.82	.49	.53
General	2.09	2.10	0.87	0.89	.77	.77	.46	.46
*Average*	*2.33*	*2.29*	*1.01*	*1.00*	*.81*	*.81*	*.53*	*.52*
FTA	2.57	2.58	0.74	0.70	.93	.92	.34	.31
LFT	2.86	2.87	0.72	0.76	.91	.92	.34	.36
CESD-R	1.93	1.95	0.58	0.56	.92	.92	.37	.36
Aggression	1.86	1.84	0.70	0.72	.84	.86	.38	.40

Aggression, Bryant Aggression Questionnaire; CESD-R, Center for Epidemiologic Studies Depression Scale – Revised; LFT, Lack of Follow Through Scale; S1, Sample 1; S2, Sample 2; CIBS, Contexts of Impulsive Behavior.

### Contexts of Impulsive Behaviors characteristics and predictive validity

**Figure 1:**
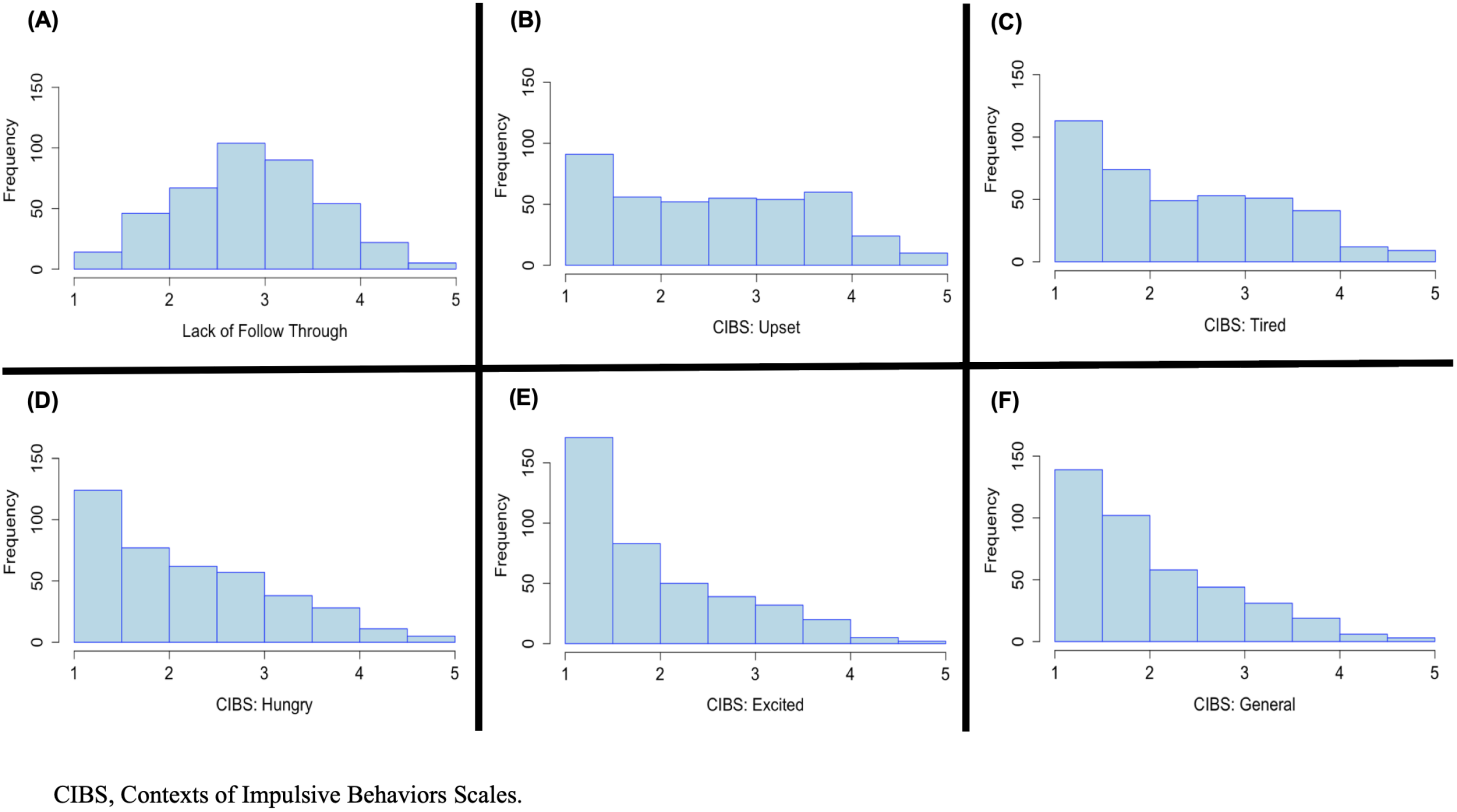
Histograms depicting the univariate distributions of **(A)** Lack of Follow Through, and the five Contexts of Impulsive Behaviors (CIBS): **(B)** Upset, **(C)** Tired, **(D)** Hungry, **(E)** Excited, and **(F)** General in Study 2.

Context-level means and standard deviations across the five CIBS contexts are reported in **Table 5**. In contrast to LFT, which was normally distributed, all five CIBS contexts were right skewed (**Figure 1**). Visual inspection of these distributions indicated that it was relatively rare for participants to score at the extreme high end of any of the contexts, as expected. Although it was common for participants in these samples to report moderate levels of LFT or impulsivity in response to feeling upset, most participants endorsed low levels across the CIBS. Tendencies to act impulsively were particularly uncommon when feeling excited. In both Sample 1 and Sample 2, reports of impulsive behaviors captured by the CIBS were most common when Upset, followed by Tired, Hungry, General, and Excited. This rank-order correlation of 1.0 provides evidence of replication for this pattern of relative commonality across the contexts we studied. Consistent with prior meta-analytic work on Urgency (63), we found small mean-level differences in the CIBS between men and women (Cohen's *d*: -0.21 – 0.20, Women > Men defined as a positive value).

Paired *t* tests demonstrated that the mean of LFT was greater than Upset, the highest CIBS mean. All adjacent CIBS means were significantly different from one another; however, Cohen's *d* was between 0.10 and 0.28, meaning that sizes of these differences were small. These results replicated in Sample 2 (**Table 6A**). Paired Pitman-Morgan tests demonstrated that LFT had less variance than General, the lowest CIBS variance. Within the CIBS, variance comparisons followed a similar order to comparisons of the means. Upset had the highest variance, followed by Tired, Hungry, Excited, and General. However, unlike the comparisons of CIBS means, none of the adjacent comparisons of CIBS sample variances were significantly different in both Sample 1 and Sample 2 (**Table 6B**). Our efficient testing method of adjacent comparisons was not sensitive to the possibility of significant differences in non-adjacent comparisons.

**Table 6:** Comparing the means and variances of adjacent Contexts of Impulsive Behaviors and Lack of Follow Through.

Panel A. Comparisons of means using paired samples t tests and effect sizes as Cohen’s d values.				
	Cohen’s *d*		95% Confidence Interval	
	S1	S2	S1	S2
Upset > Tired	0.23	0.26	0.13 – 0.33	0.16 – 0.36
Tired > Hungry	0.22	0.19	0.12 – 0.32	0.09 – 0.29
Hungry > General	0.28	0.20	0.18 – 0.38	0.10 – 0.30
General > Excited	0.10	0.14	0.00 – 0.20	0.04 – 0.24
Upset > LFT	0.15	0.20	0.05 – 0.25	0.11 – 0.30
Panel B. Comparisons of variances using paired Pitman-Morgan tests.				
	s^2^_1_, s^2^_2_		95% Confidence Interval	
	S1	S2	S1	S2
Upset > Tired	1.32, 1.16	1.22, 1.12	0.99 – 1.32	0.95 – 1.26
Tired > Hungry	1.16, 1.07	1.12, 1.01	0.94 – 1.24	0.95 – 1.29
Hungry > Excited	1.07, 0.82	1.01, 0.87	1.11 – 1.55	0.98 – 1.38
Excited > General	0.82, 0.76	0.87, 0.78	0.93 – 1.25	0.95 – 1.28
General > LFT	0.76, 0.52	0.78, 0.58	1.22 – 1.72	1.14 – 1.60

LFT, Lack of Follow Through Scale; S1, Sample 1; S2, Sample 2; s^2^_1_, variance of variable 1; s^2^_2_, variance of variable 2.

All impulsivity and psychopathology measures used in this analysis formed continuous distributions, so we used Pearson's correlations to test the strengths of bivariate effects. CIBS contexts were highly intercorrelated in both Sample 1 and Sample 2, with Pearsons *r* ranging from 0.52 – 0.70, all *p*s <.001 (**Table 7**). Of interest, CIBS Upset and CIBS Excited, the two emotion-related contexts, were not more highly correlated with one another than they were with the other three CIBS contexts, as indicated by overlapping 95% confidence intervals. As expected, correlations of CIBS and FTA were large with Pearson's *r* ranging from 0.62 – 0.79 and stronger than correlations of CIBS and LFT with Pearson's *r* ranging from 0.36 – 0.49, as indicated by non-overlapping 95% confidence intervals.

**Table 7:** Zero-order correlations (Pearson’s *r*) and 95% confidence intervals among the Contexts of Impulsive Behaviors and Lack of Follow Through scale.

	Upset	Tired	Hungry	Excited	General	FTA	LFT
Upset	–						
Tired	.69 [.63-.73] .68 [.62-.73]	–					
Hungry	.58 [.51-.64] .63 [.57-.69]	.70 [.65-.75] .64 [.58-.70]	–				
Excited	.58 [.51-.64] .56 [.49-.63]	.59 [.52-.65] .65 [.59-.70]	.53 [.45-.59] .52 [.44-.59]	–			
General	.67 [.61-.72] .70 [.64-.74]	.65 [.59-.70] .69 [.63-.73]	.63 [.57-.68] .62 [.56-.68]	.67 [.62-.72] .64 [.58-.70]	–		
FTA	.67 [.61-.72] .73 [.68-.77]	.65 [.59-.70] .67 [.61-.72]	.63 [.56-.68] .62 [.56-.68]	.63 [.56-.68] .62 [.56-.68]	.79 [.74-.82] .75 [.70-.79]	–	
LFT	.41 [.32-.49] .42 [.34-.50]	.36 [.27-.44] .44 [.35-.51]	.39 [.30-.47] .37 [.28-.45]	.37 [.28-.45] .38 [.30-.46]	.49 [.42-.56] .48 [.40-.56]	.48 [.40-.55] .49 [.41-.56]	–

Sample 1 (top) and Sample 2 (bottom); FTA, Feelings Trigger Action; LFT, Lack of Follow Through.

All bivariate correlations of CIBS with psychopathology indices were of moderate to high strength, *r*s = .24 to.47, (*p* < 0.001; **Table 8A**). Our key comparisons of the bivariate effects of emotion versus broader physiological CIBS contexts on psychopathology indicated no significant differences. That is, as shown in **Table 8A**, the 95% confidence intervals around all the bivariate effects of the five CIBS contexts on psychopathology overlapped one another. In contrast to our prediction, CIBS contexts did not have stronger bivariate correlations with depression symptoms than LFT did. The effect of CIBS General on aggression was significantly larger than the effect of LFT, but comparisons of other CIBS contexts and LFT were null.

**Table 8:** Pearson’s *r* and 95% confidence intervals for zero-order and partial effects of the Contexts of Impulsive Behaviors with depression and aggression.

Panel A. Zero-Order Correlations of the Contexts of Impulsive Behaviors and the Lack of Follow Through (LFT) Scale with Psychopathology							
* *		Upset	Tired	Hungry	Excited	General	LFT
CESD-R	S1	.38 [.29-.46]	.43 [.34-.50]	.39 [.30-.47]	.36 [.27-.44]	.47 [.39-.54]	.50 [.43-.57]
	S2	.30 [.21-.39]	.32 [.23-.41]	.28 [.19-.37]	.26 [.17-.35]	.43 [.35-.51]	.47 [.39-.54]
Aggression	S1	.37 [.28-.45]	.39 [.30-.47]	.35 [.26-.43]	.37 [.28-.45]	.47 [.39-.55]	.27 [.18-.36]
	S2	.31 [.21-.39]	.31 [.22-.39]	.24 [.15-.33]	.32 [.23-.41]	.42 [.34-.50]	.24 [.14-.33]
Panel B. Partial Correlations of the Emotion-Related Contexts of Impulsive Behaviors and Psychopathology, Controlling for Tired and Hungry							
* *		Upset			Excited		
CESD-R	S1	.11 [.01-.20]			.13 [.03-.23]		
	S2	.09 [-.01-.19]			.05 [-.05-.15]		
Aggression	S1	.13 [.04-.23]			.17 [08.-.27]		
	S2	.13 [.03-.22]			.16 [.07-.26]		
Panel C. Partial Correlations of the Tired and Hungry Contexts of Impulsive Behaviors and Psychopathology, Controlling for Upset and Excited							
* *		Tired			Hungry		
CESD-R	S1	.20 [.11-.29]			.19 [.09-.28]		
	S2	.14 [.04-.23]			.10 [.00-.19]		
Aggression	S1	.15 [.05-.24]			.13 [03.-.22]		
	S2	.06 [-.04-.16]			.02 [-.08-.12]		
Panel D. Partial Correlations of Contexts of Impulsive Behaviors and Psychopathology, Controlling for General							
* *		Upset	Tired		Hungry	Excited	
CESD-R	S1	.10 [-.00-.19]	.18 [.08-.27]		.14 [.04-.23]	.07 [-.03-.17]	
	S2	.00 [-10.-.10]	.04 [-.06-.14]		.02 [-.08-.12]	-.03 [-.13-.07]	
Aggression	S1	.08 [-.02-.18]	.12 [.02-.21]		.07 [-.02-.17]	.08 [-.02-.18]	
	S2	.02 [-.08-.12]	.03 [-.07-.13]		-.02 [-.12-.07]	.08 [-.02-.17]	
Panel E. Partial Correlations of the Contexts of Impulsive Behaviors and Psychopathology, Controlling for Lack of Follow Through							
* *		Upset	Tired	Hungry	Excited	General	
CESD-R	S1	.22 [.13-.31]	.31 [.21-.39]	.25 [.15-.34]	.23 [.13-.32]	.30 [.21-.39]	
	S2	.13 [.04-.23]	.15 [.06-.25]	.13 [.04-.23]	.10 [-.00-.19]	.27 [.17-.36]	
Aggression	S1	.30 [.20-.38]	.33 [.23-.41]	.28 [.18-.36]	.30 [.21-.39]	.41 [.32-.49]	
	S2	.23 [.14-.32]	.23 [.14-.32]	.17 [.08-.27]	.26 [.17-.35]	.36 [.27-.44]	

Sample 1 (top) and Sample 2 (bottom); Aggression, Bryant Aggression Questionnaire; CESD-R, Center for Epidemiologic Studies Depression Scale – Revised.

**Tables 8B–D** display the results from partial correlations testing unique effects of CIBS on psychopathology. Overall, the effects of the CIBS emotion contexts on psychopathology were attenuated when controlling for broader physiological CIBS contexts (**Table 8B**). Though the Upset context accounted for unique depression variance in Sample 1 when controlling for the Tired and Hungry contexts, this unique effect was not significant in Sample 2. The same pattern across Samples 1 and 2 was found when examining the unique effect of the Excited context on depression when controlling for the Tired and Hungry contexts; that is, the Excited context accounted for unique depression variance in Sample 1 but not Sample 2. Unique effects of emotion-context impulsivity on aggression were more robust. Across both samples, the Upset and Excited contexts each accounted for unique variance in aggression when controlling for the Tired and Hungry contexts.

Going beyond the pre-registered analyses, we examined the unique effects of CIBS Tired and Hungry contexts when controlling for the CIBS emotion contexts (**Table 8C**). Mirroring the partial correlations above, the correlations of CIBS Tired and Hungry on psychopathology were partially or fully attenuated when controlling for the CIBS emotion contexts. Across both samples, the Tired and Hungry contexts accounted for unique variance in depression when controlling for the emotion contexts. The Tired and Hungry contexts accounted for unique variance in aggression in Sample 1, but not in Sample 2.

We also examined the unique effects of CIBS emotion and broader physiological contexts on psychopathology when controlling for the CIBS General item set (**Table 8D**) and LFT (**Table 8E**). In both Sample 1 and Sample 2, the General item set fully attenuated the effects of the Upset and Excited contexts on depression and aggression. The Tired and Hungry contexts explained unique variance in depression when controlling for the General item set in Sample 1, but not in Sample 2. Across 10 partial correlations, the unique effects of CIBS on depression and aggression remained significant when controlling for LFT, with one exception. When controlling for LFT, the Excited context accounted for unique variance in depression scores in Sample 1, but not in Sample 2.

## Discussion

The primary aim of these two studies was to adjudicate among two competing models of urgency: the Emotion Specific model, which highlights the uniqueness of emotional states as contexts for impulsive behavior, in contrast to the Broader Contexts model, which posits that broader physiological factors, might prompt impulsive behavior. Our results begin to address a key question in the impulsivity literature: Are emotions a special class of contexts for impulsive behavior, and therefore a necessary component of the urgency construct – as predicted by the Emotion Specific model – or does this trait reflect less specific susceptibility to a range of state-level regulatory challenges, consistent with the Broader Contexts model? We conducted several pre-registered analyses to evaluate the relative merits of these alternative frameworks.

First, we conducted PCA of self-report items pertaining to contextual impulsivity in a discovery sample, which yielded an internally reliable and unidimensional four-item solution. This four-item solution was replicated in a second sample with similarly strong reliability and fit on single factors within each of our specified contexts – Upset, Tired, Hungry, and Excited, as well as a General item set that captured the urgency-like rash action without reference to contextual antecedents. We refer to the empirically-derived composite of these four-probe item sets as the “Contexts of Impulsive Behaviors” (CIBS). Consistent with the idea that LFT and Urgency measure distinct phenomena that contribute separate but overlapping variance to psychopathology (64, 65), the CIBS contexts only moderately correlated with LFT and demonstrated distinct distributions across two well-powered adult samples. In service of our primary aim to understand the specificity of impulsive responses, we have constructed this very brief, 4-item impulsive behavior research tool that was not only highly reliable but also strictly unidimensional upon replication, both in general and for 4 contexts (upset, tired, hungry, and excited).

Although the Emotion Specific model was supported by differences in the distribution of the Upset context, which had a larger mean relative to the Tired and Hungry contexts, most analytic results aligned with the Broader Contexts model. Compared to scores on the Excited context, means and variances of the Tired and Hungry contexts were more similar to the Upset context. That is, people were more likely to report that they engaged in rash action when they were tired or hungry than when they were excited. CIBS contexts were also highly intercorrelated, to the extent that they approached maximum possible effect sizes allowed by the internal reliability of these measures. Perhaps most importantly, emotion, hunger, and tiredness had statistically equivalent bivariate effects on internalizing and externalizing psychopathology. Not only were the effects on psychopathology comparable, but there was little evidence for unique, independent pathways. Partial correlations indicated (partial or full) statistical mediation of the Upset and Excited contexts effects on psychopathology by scores on (a) the Tired and Hungry contexts as well as (b) the General item set. Correlations between CIBS scores and psychopathology were nominally higher in Sample 1 than in Sample 2, yet the rank order of means and variances and the overall patterns of effects across contexts were highly consistent.

Overall, we found that individuals with high levels of Urgency tended to endorse vulnerability to varied contextual challenges to self-regulation. Although emotion – particularly in the negative valence domain – will certainly prompt rash action among many people, most of these individuals will also struggle with impulsive behavior secondary to hunger and tiredness. These findings do not override the importance of emotion as a key prompt of disinhibition. That is, intense emotions remain clear and potent prompts for impulsive behavior captured by Urgency measures; however, just as the urgency construct was previously expanded to include positive emotional contexts (53), these two studies provide evidence that supports revising theoretical models of urgency to further broaden its scope.

Although emotion, hunger, and fatigue may seem qualitatively distinct, we chose these contexts because each has been shown to influence circuitry involved in inhibitory control. Therefore, our findings are consistent with the idea that urgency may reflect a broader fragility of inhibitory control circuitry, which is unimpaired under ideal conditions, but becomes overwhelmed under conditions of high physiological arousal or depleted cognitive resources. Caution is warranted though, as we were not able to test inhibitory control directly in this study and so have no direct evidence of mechanisms. Indeed, one possibility is that hunger and exhaustion lead to greater emotionality, which then is reflected in impulsive behavior.

Despite obtaining results consistent with the Broader Contexts model of urgency, there may be value to retaining conceptually dissociable constructs (and accompanying measures) of trait impulsivity in emotion- and broader physiological-contexts. While the observed intercorrelations among CIBS scores were substantial, there remained important differences in the means and variability of the subscale distributions. From a practical perspective, there is clinical utility to examining contexts of problematic behavior more granularly, for example, to develop personalized intervention strategies specific to a clients particular pattern of dysregulation. Also of ecological relevance, some people are likely to experience certain types of contexts more frequently due to structural factors inherent to specific environments or circumstances (e.g., shift work). Fortunately, one positive implication of this work is the idea that interventions targeting urgency may be more broadly efficacious (e.g., emotion regulation skills & implementation intentions; 61, 66). For people who struggle with emotion as a precipitant for undesirable behavior, therapists might do well to consider other contexts known to challenge inhibitory control, to ideographically map key contexts for each client. Together, these findings are consistent with lay conceptualizations of impulsive acts involving diverse antecedents, including but not limited to emotions – for instance, as evidenced by widespread use of the term “hangry” (a portmanteau of hungry and angry) to explain disinhibited behavior, or “cranky”, which is often used to indicate poor control in the face of exhaustion.

Researchers have long assumed that emotion dysregulation is the core concept underlying the explanatory power of Urgency scales, which beautifully integrate the interactive influences of emotion and self-control on behavioral outcomes. Our findings, in which we used the probes without a specific context (“in general”) though, suggest the Urgency scales might also perform well in predicting clinical outcomes due to the consequences of the forms of poor constraint covered, e.g., making regrettable choices, speaking inappropriately, etc. That is, we found that when we asked participants to rate their tendencies using the CIBS item probes “in general”, the resultant scores were as or more powerfully related to psychopathology than were the context-specific subscales. This may come as a surprise, since Urgency items *sans* emotion resemble the Lack of Premeditation scale from the UPPS (i.e., acting without thinking), and Lack of Premeditation typically has smaller correlations with psychopathology than Urgency does (6). One important distinction is that the CIBS General (i.e., urgency *sans* emotion) still contains the themes of “regret,” “making matters worse,” and “consequences,” and the Lack of Premeditation items do not. Although we are not equipped to test for separability of the CIBS General and the Lack of Premeditation scale in this study, we see this as an intriguing future direction for those interested in assessing the “active ingredients” of the urgency construct, as it pertains to its robust predictive validity.

Several limitations should be considered when interpreting these two studies. Contrary to expectations and prior work, depression (as evaluated by the CESD-R) was not more strongly related with Urgency than LFT. Here, it is worth noting that previous research has shown that current depression symptoms exert deleterious impact on processes implicated in LFT, including attention, motivation, and persistence (67, 68). This highlights the importance of longitudinal studies. One such study found that Urgency was a stronger long-term predictor of depression than LFT; however, more work is needed to disentangle cognitive impacts of current depression from those captured by Urgency and LFT (69).

Beyond the unexpectedly large effect of LFT on depression, generalizability of our results might be constrained by narrow representation in the recruited undergraduate samples. Notably, with escalating levels of depression among young persons, rates of psychiatric syndromes in college students now approximate those in the general population (70). Indeed in considering generalizability, it is important to note that these data were gathered during the COVID-19 pandemic. Although participants had returned to mostly in-person classes at the time of their participation in the current studies, trait impulsivity correlated with increased internalizing symptoms during the COVID-19 pandemic (71, 72). The relative magnitudes of the effect sizes found with internalizing symptoms in this study could have been influenced by the fact that the impacts of the COVID-19 pandemic were still very proximal.

Beyond sample characteristics, our data also rely on retrospective self-report measures, and are thus prone to subjective (e.g., interpretive, recall) biases inherent to such assessment modalities. For example, retrospective urgency measures might blur distinctions among various contexts of past impulsive behavior or the temporal order of impulsivity-relevant antecedents and consequences. Future work in this area should also account for potential influences of social desirability, e.g., the perceived acceptability of impulsive acts across different contexts.

Our findings suggest directions for future research. Although we provide evidence that the validity of impulsivity assessment may be improved by inquiring about a greater variety of precipitants to rash actions, the contexts studied here (emotions, hunger, and fatigue) are not exhaustive. Among the many potentially important contexts, intriguing research suggests that stress may have unique effects compared to mood inductions on inhibitory processes, and we did not consider stress here (73–75). More research is needed to understand which physiological contexts do and *do not* prompt the trait-like tendency toward impulsive action traditionally captured by the Urgency scale. By studying points of overlap on this more detailed contextual topography, future researchers may be able to generate more comprehensive models of urgency.

The current work is also limited in the range of psychopathology dimensions studied. Future work might assess a broader range of psychopathologies, and particularly those that have been shown to be uniquely tied to Positive Urgency in previous studies, such as alcohol use disorder (25) and mania (76). With this, it will be important to consider that particular contextual factors may confer greater vulnerability for certain individuals (perhaps especially at specific points in time), e.g., hunger in food-insecure populations. Ecological momentary assessment (EMA) may be an especially promising approach to explore these proposed future directions. EMA would address memory biases and facilitate temporal disambiguation of apparently concomitant precipitants of dysregulation (e.g., see 77, 78). Perhaps most importantly, EMA work would enable person-specific modeling that could ultimately inform clinical intervention and prevention efforts. Intriguing EMA findings from Sharpe et al. (79) suggest, for example, that within-person models might provide the strongest evidence for Negative and Positive Urgency as dissociable processes and constructs. Finally, there are multiple competing models of how contextual forces may influence disinhibition, including the depleted cognitive resource model (19, 80), the neurobiological salience model (81, 82), and the integrated model of stress, executive control, and psychopathology (75). Broadening the focus to an array of contexts while directly assessing inhibitory control and attentional circuitry and performance may help in considering the relative contributions of these types of processes.

In sum, we interpret these findings to suggest that people with elevated Urgency are likely vulnerable to various sources of disrupted self-regulation, beyond intense affect. Rather than de-emphasize the importance of emotion-related impulsivity to psychopathology, we argue that results of our pre-registered analyses justify expanding this construct to more fully capture the diversity of contextual influences on inhibitory failures that produce impulsive action. This new perspective ought to guide researchers to consider a broader range of forces that might adversely influence prefrontal inhibitory control mechanisms (and executive functioning in general).

With replication and extension, we see these findings as having clinical implications as well. Overall, they highlight the need for clinicians to consider a broader range of contexts for impulsive behaviors. Interventions such as Dialectical Behavior Therapy provide techniques for coaching clients to consider both physiological and emotional antecedents to behaviors of concern, and so might be particularly helpful in light of this broader set of possible contexts to consider (83, 84). We hope the findings will also guide clinicians to consider interventions that build resilience to a wider array of contexts that commonly prompt impulsivity.

## Data Availability

The datasets presented in this study can be found in online repositories. The names of the repository/repositories and accession number(s) can be found below: ResearchBox (#2750): https://researchbox.org/2750&PEER_REVIEW_passcode=MQDIIC.
