# Supplementary material for: Contexts of urgency may go beyond emotion
Source: Front Psychiatry. 2024 Sep 12;15:1412001. doi: 10.3389/fpsyt.2024.1412001 (PMC11424539; doi:10.3389/fpsyt.2024.1412001)
Supplement: Supplementary file 1 [file Table1.docx]

*Appendix A. Initial Item Set to Measure Broader Contexts of Impulsivity.*

Please rate how true the following statements are for your life in general.

- 1. I often make matters worse because I act without thinking. [NU]
  2. I will often do things I later regret in order to make myself feel better now. [NU]
  3. I do not have trouble controlling my impulses. (R) [NU]
  4. I have trouble resisting my cravings (for food, cigarettes, etc). [NU]
  5. I will often say things that I later regret. [NU]
  6. Others are shocked or worried about the things I do. [PU]
  7. I think of the consequences of my actions. (R) [PU]
  8. I feel like I can’t stop myself from going overboard. [PU]
  9. I can’t seem to stop what I am doing even though it is making me feel worse. [NU]

Now, we want you to think about times when you are feeling upset. Please rate how true the following statements are for you when you are upset.

- 1. I often make matters worse because I act without thinking.
  2. I will often do things I later regret in order to make myself feel better now.
  3. I do not have trouble controlling my impulses. (R)
  4. I have trouble resisting my cravings (for food, cigarettes, etc).
  5. I will often say things that I later regret.
  6. Others are shocked or worried about the things I do.
  7. I think of the consequences of my actions. (R)
  8. I feel like I can’t stop myself from going overboard.
  9. I can’t seem to stop what I am doing even though it is making me feel worse.

Now, we want you to think about times when you are feeling very excited. Please rate how true the following statements are for you when you are very excited.

- 1. I often make matters worse because I act without thinking.
  2. I will often do things I later regret in order to make myself feel better now.
  3. I do not have trouble controlling my impulses. (R)
  4. I have trouble resisting my cravings (for food, cigarettes, etc).
  5. I will often say things that I later regret.
  6. Others are shocked or worried about the things I do.
  7. I think of the consequences of my actions. (R)
  8. I feel like I can’t stop myself from going overboard.
  9. I can’t seem to stop what I am doing even though it is making me feel worse.

Now, we want you to think about times when you have not had enough sleep. Please rate how true the following statements are for you when you have not had enough sleep.

- 1. I often make matters worse because I act without thinking.
  2. I will often do things I later regret in order to make myself feel better now.
  3. I do not have trouble controlling my impulses. (R)
  4. I have trouble resisting my cravings (for food, cigarettes, etc).
  5. I will often say things that I later regret.
  6. Others are shocked or worried about the things I do.
  7. I think of the consequences of my actions. (R)
  8. I feel like I can’t stop myself from going overboard.
  9. I can’t seem to stop what I am doing even though it is making me feel worse.

Now, we want you to think about times when you are feeling hungry. Please rate how true the following statements are for you when you are hungry.

- 1. I often make matters worse because I act without thinking.
  2. I will often do things I later regret in order to make myself feel better now.
  3. I do not have trouble controlling my impulses. (R)
  4. I have trouble resisting my cravings (for food, cigarettes, etc).
  5. I will often say things that I later regret.
  6. Others are shocked or worried about the things I do.
  7. I think of the consequences of my actions. (R)
  8. I feel like I can’t stop myself from going overboard.
  9. I can’t seem to stop what I am doing even though it is making me feel worse.

*Appendix B. Loadings of the 9 Candidate Items on the First Three Unrotated Principal Component in the Hungry Context in Sample 1.*

|  |  |  |  |
| --- | --- | --- | --- |
| Item | PC1 | PC2 | PC3 |
|  |  |  |  |
| Items Showing Simple Structure | |  |  |
| Item 2 | 0.82 | 0.03 | -0.08 |
| Item 1 | 0.82 | -0.11 | -0.01 |
| Item 5 | 0.74 | -0.27 | 0.13 |
| Item 7 | 0.79 | -0.08 | 0.16 |
| Item 9 | 0.76 | -0.01 | -0.09 |
|  |  |  |  |
| Factorially Complex Items | |  |  |
| Item 6 | 0.66 | -0.27 | 0.41 |
| Item 3 | 0.49 | 0.55 | -0.28 |
| Item 4 | 0.54 | 0.30 | -0.50 |
| Item 8 | 0.11 | 0.73 | 0.64 |
|  |  |  |  |

Note. PC1 to PC3 are the first three unrotated principal components. The first five items listed here all showed clear simple structure: they all loaded above .70 (shown in bold) on the first principal component and none had a loading of .35 or above on the other two components. The remaining items were factorially complex: They had loadings below .70 on the first principal component and substantial loadings (shown in italics) on at least one of the two other components.
